# Identification of new cytotypes of *Valeriana jatamansi* Jones, 1970 (Valerianaceae) from North-Western Himalayan region of India

**DOI:** 10.3897/CompCytogen.v9i4.8875

**Published:** 2015-08-07

**Authors:** Savita Rani, Tilak Raj Sharma, Rakesh Kapila, Rakesh Kumar Chahota

**Affiliations:** 1 Department of Agricultural Biotechnology, CSK HPKV Palampur (HP) 176 062 India

**Keywords:** *Valeriana jatamansi*, cytotypes, meiotic abnormalities, morphovariants, western Himalayas

## Abstract

*Valeriana jatamansi*, a medicinally important species of the family Valerianaceae, has been cytologically studied in different geographical areas of North-Western Himalayan region of India. The tetraploid cytotype with chromosome numbers 2n=32 is in conformity with the earlier reports of the species from different parts of the world. An octoploid cytotype (2n=64) makes a new addition for the species on a worldwide basis, whereas the diploid cytotype (2n=16) is new to India have been reported for the first time in India. These cytotypes (2n=16, 32, 64) show significant variations with respect to morphology as well as geographical distribution in the Western Indian Himalayas. Further, anomalous populations have been marked with meiotic abnormalities in the form of cytomixis, chromosomal stickiness, unoriented bivalents, formation of laggards and bridges resulting in abnormal microsporogenesis, and production of heterogeneous-sized fertile pollen grains along with reduced pollen fertility.

## Introduction

The genus *Valeriana* Linnaeus, 1753 belongs to the family Valerianaceae which comprises 250 species of perennial herbs (Chen et al. 1999) distributed throughout temperate and cold regions of the Northern hemisphere (Bell 2004). In India, 16 species and two subspecies of the genus have been reported; of these 5 species inhabit the high-altitude ranges of the Kumaon and Garhwal regions of central Himalayas (Prakash 1999).

*Valeriana jatamansi* Jones (= *Valeriana wallichii* de Candolle, 1830) popularly known as Indian valeriana (English), Mushkibala (Kashmiri/ Hindi), Sugandhwala or Tagar (Sanskrit), grows wild in the temperate Himalayan region between 1000 and 3000 m altitude. The species generally grows on sloppy, moist places, damp woods, ditches and along the streams. The species grows particularly in the understory of *Quercus leucotrichophora* Camus, 1938 – *Pinus roxburghii* Sargent, 1897 mixed forests and on grassy habitats of Himalayas. Its occurrence can be observed in different geographical areas and the species possesses diverse morphological and genetic features, affecting its levels of active ingredients. *Valeriana jatamansi* is a perennial herb with thick root stocks, horizontal thick descending fibers with pubescent stem, radical leaves often 1-3 cm in diameter, deeply cordate and usually acute toothed margins. Flowers white, stamens- 3, ovary -3 celled, stigma shortly 2-3 fid and fruits oblong lanceolate and compressed crowned by persistent pappus calyx. The flowering and fruiting time for the species is March–June. The mode of propagation is both sexual through seeds and asexual through rhizome.

The species is being exploited for its roots and rhizomes which contain valepotriates (Chopra 1956) highly effective against leprosy (Kour et al.1999) and curing Lewybody dementia (Bagchi and Hooper 2011). The reported annual collection of *Valeriana jatamansi* from the North-Western Himalayas is about 100 quintals whereas hundreds of quintals *Valeriana* roots are smuggled and go unnoticed. Due to over-exploitation of rhizomes for its medicinal value, it is labeled as endangered plant species in the Himalayan region. In spite of its importance as a medicinal plant, there is a lack of information regarding genetic diversity and chromosome number of different cytotypes present in the area, which is prerequisite for initiation of any systematic breeding programme.

Therefore, keeping in view the economic importance, threatened status of the species, and cytological variability within the species, at present cytomorphological studies have been carried out on a population basis from different areas of the North-Western Himalayas. The present study also discusses the impact of cytomixis on meiotic behavior and reduced pollen fertility and formation of heterogeneous-sized pollen grains in the species.

## Material and methods

**Material.** Material for cytological studies in the form of buds was collected from different parts of the North-Western Himalayas. The propagating material of these plants was also collected and planted in the experimental fields of Chaudhary Sarwan Kumar Himachal Pradesh Agricultural University, Palampur, India in a Randomized Complete Block design with two replications. The plant to plant distance was kept at 5 cm while row to row distance was kept at 50 cm.

### A) Morphological study

Different qualitative morphometric characters were studied for each cytotype to have proper insight on morphological variation present in these cytotypes. For stomatal studies, mature leaves were treated with 10% aqueous solution of potassium hydroxide (KOH) at room temperature for 10–15 min and then epidermal peels so obtained were stained with 10% saffanin in 90% ethanol. In order to reveal the significant difference in the stomata and pollen grain size of diploid, tetraploid and octaploid cytotypes, the t-test was been performed.

### B) Cytological studies

For meiotic studies, flower buds were collected from plants growing under natural conditions from selected areas of the Western Himalayas. These flower buds were collected from 15 randomly selected plants of each population and fixed in Carnoy’s fixative (6:3:1 ethanol/chloroform/acetic acid v/v/v) for 24 h. Flower buds were washed and preserved in 70% ethanol at 4 °C until used. Smears of appropriate-sized flower buds were made, using the tandard acetocarmine technique (Marks 1954). About 20–50 fresh slides in each case were prepared from different anthers/flowers for different individuals of a particular population and were analyzed in each case. To confirm the chromosome number in case of normal meiosis, around 50 pollen mother cells (PMCs) were observed at different stages of meiosis, preferably at diakinesis/ metaphase-I/ anaphase-I, II. In case of abnormal meiosis, however, more than 300 PMCs were considered to ascertain the type and frequency of various abnormalities per plant. Pollen fertility was estimated by mounting mature pollen grains in glycero–acetocarmine (1:1) mixture (Belling 1921). Nearly 400–500 pollen grains were analyzed in each case for ascertaining pollen fertility and pollen size. Well-filled pollen grains with stained nuclei were taken as apparently fertile, while shrivelled and unstained pollen grains were counted as sterile. Photomicrographs of pollen mother cells and pollen grains were made from freshly prepared slides using Optika Digital Imaging System. The data regarding the number of cytologically worked out species, number of cytotypes, and frequency of polyploids of a particular genus have been compiled on worldwide and India basis from various Chromosomal Atlases and Indexes to Plant Chromosome Numbers by Darlington and Wylie (1955), Fedorov (1974), Moore (1973, 1974, 1977), Kumar and Subramanian (1986), and Khatoon and Ali (1993), various journals, Internet resources, as well as presently studied plants.

## Results and discussion

Detailed meiotic studies were carried out on 22 populations of *Valeriana jatamansi* collected from different localities with altitude ranging from 764 to 3647 m above mean sea level in the North-Western Himalayan region of India. The data regarding locality with altitude, latitude and longitude, present meiotic chromosome number, ploidy level and meiotic course of the presently worked out populations have been presented in Table 1.

**Table 1.** Information about, locality, latitude and longitude, altitude, meiotic chromosome number, ploidy level and meiotic course of *Valeriana jatamansi*.

S. No.	Locality with latitude and longitude, altitude	Present meiotic chromosome number (2n)	Ploidy level	Meiotic in meters
**District Chamba (Himachal Pradesh)**				
P-1	Bhali mata/ 32°37'N, 76°0’E/1,900	32	4*x*	Abnormal
P-2	Salooni/32°43'N,76°03'E/1,900	16	2*x*	Normal
P-3	Chamba/ 32°33'N, 76°07'E/ 1,200	32	4*x*	Abnormal
P-4	Leg Valley/31°58'N, 77°06'E/ 1,720	32	4*x*	Normal
P-5	Tisa/32°32'N, 76°08'E/ 1,220	32	4*x*	Normal
P-6	Dehgram/32°41'N, 76°08'E/ 2,165	32	4*x*	Abnormal
**District Shimla (Himachal Pradesh)**				
P-7	Kandi/32°36'N, 76°02'E/ 854	32	4*x*	Normal
P-8	Rampur/ 31°58'N,77°06'E/ 1,350	16	2*x*	Normal
P-9	Pander/ 31°26'N,77°03'E/ 2,236	32	4*x*	Normal
P-10	Shimla I/ 31°6'N, 77°10'E/ 2,202	32	4*x*	Abnormal
P-11	Shimla II/31°18'N, 77°20'E/ 1,820	32	4*x*	Normal
**District Mandi (Himachal Pradesh)**				
P-12	Rewalsar/31°38'N, 76°50'E/ 1,360	32	4*x*	Normal
P-13	Rakni/ 31°24'N, 77°07’E/1,649	32	4*x*	Normal
P-14	Prashar / 31°45'N, 77°6'E / 2,200	32	4*x*	Abnormal
P-15	Mandi I/ 31°42'N, 76°51'E/ 764	32	4*x*	Normal
P-16	Mandi II/31°31'N, 76°59'E/ 945	32	4*x*	Abnormal
**District Kullu (Himachal Pradesh)**				
P-17	Kullu I/ 32°09'N,77°02'E/ 3,647	64	8*x*	Abnormal
P-18	Sojha/31°42'N, 77°54'E/ 2,692	32	4*x*	Normal
P-19	Jalori Pass/ 31°32'N, 77°22'E/ 3,134	64	8*x*	Normal
P-20	Kullu II/ 32°08'N, 77°04'E/ 2,734	32	4*x*	Abnormal
P-21	Parvati valley/32°01'N, 77°20'E/1,640	32	4x	Normal
P-22	Manikaran /32°08'N, 77°26'E/ 1,820	32	4x	Normal

### A) Morphological observations

**Figure 1.**
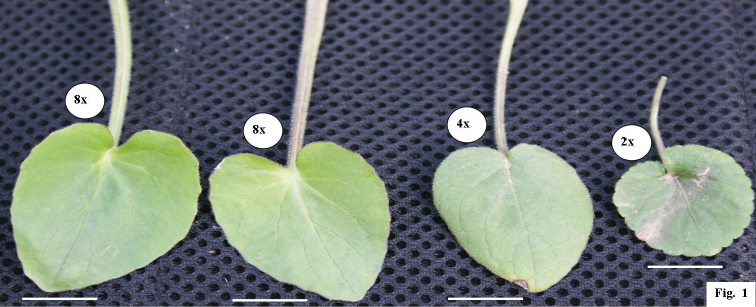
Leaves showing variations among 2x, 4x and 8x cytotypes of *Valeriana jatamansi*.

Morphological variation was assessed on 24 vegetative and reproductive characters of different cytotypes of *Valeriana jatamansi* (Table 2). All the three cytotypes (2x, 4x and 8x) at intraspecific level revealed significant variations for some of the qualitative characters as is evident from increased stomatal size, guard, and subsidiary cells, stomatal frequency and index in polyploids as compared to diploids (Table 2). Along with these characters, some significant differences were noticed for leaf size in all the three cytotypes (Fig. 1). The size of pollen grain in the octaploid was also found to be significantly larger than their diploid equivalent (p<0.05, Table 2). Such comparable results have been previously reported for many angiosperms such as in *Rorippa amphibia* Besser, 1822 (Luttikhuizen et al. 2007), *Nicotiana alata* Link & Otto, 1840 (El-Morsy et al. 2009), *Ocimum basilicum* Linnaeus, 1753 (Omidbaigi et al. 2010), etc. These morphological variations may be attributed to the variation in chromosome number as has been reported earlier in *Centaurea stoebe* Ledebur, 1833 (Nakagawa 2006 and Španiel et al. 2008), *Chamerion angustifolium* (Linnaeus, 1753), *Heuchera grossulariifolia* Rydberg, 1900, *Vaccinium corymbosum* Linnaeus, 1753 (Soltis et al. 2007), *Ranunculus parnassifolius* Linnaeus, 1753 (Cires et al. 2009), *Ranunculus hirtellus* Royle, 1753 (Kumar 2011), etc. Overall, increase in ploidy level is correlated with gigantism for some of the vegetative and floral characters.

**Table 2.** Detailed morphological comparison of three cytotypes of *Valeriana jatamansi*.

No.	Morphological character	Diploid (2n=16)	Tetraploid (2n=32)	Octaploid (2n=64)
**1**	Distribution	Uncommon	Most Common	Uncommon
**2**	Habit	Small sized herb	Medium sized herb	Medium sized herb
**3**	Habitat	Found under shade of *Pinus roxburghii*	Found in the moist, shady and humus rich places	Found on the slopes in forests
**4**	Plant height (cm)	14.42±2.84	19.46±2.34	28.70±1.69
**Stem**				
**5**	Surface	Non-glabrous	Non-glabrous	Glabrous
**6**	No. of hairs/ mm^2^	-		12.6±1.3
**7**	Length of hairs (cm)	-	-	2.3±0.38
**Basal Leaves**				
**8**	Number of leaves / plant	22.66±7.76	20.77±4.55	47.50±10.60
**9**	Shape	Ovate	Lanceolate	Lanceolate
**10**	Size (cm)	4.53±0.95×5.83±1.05	5.56±0.86×6.98±1.01	7.65±0.63×7.26±1.36
**11**	Surface	Non-glabrous	Non-glabrous	Glabrous
**12**	Leaves Margin	Toothed	Entire	Wavy
**Cauline Leaves**				
**13**	Number of leaves / plant	4.00±1.00	11.41±2.18	21±2.41
**14**	Size (cm)	4.23±0.70×4.63±0.64	6.53±0.59×7.40±0.74	7.41±2.18×21.00±1.41
**15**	Surface	Non-glabrous	Non-glabrous	Glabrous
**16**	Leaves Margin	Toothed	Entire	Entire
**Stomata**				
**17**	Size (µm)	21.66±1.56×18.3±0.36	23.82±0.76×19.58±0.67	26.74±0.35×20.55±0.48
**18**	Stomatal frequency on upper/ lower surface of leaf (mm^2^)	5.41±0.45/3.25±0.36	5.48±0.48/7.45±0.45	3.25±0.36/3.78±0.11
**19**	Stomatal index of upper/ lower surface of leaf (µm)	22.28/12.45	23.45/13.24	24.50/14.32
**Inflorescence**				
**20**	Length (cm)	9.9±1.8	10.2±3.5	10.7±1.8
**21**	Diameter (cm)	2.69±0.23	2.39±0.28	2.34±0.30
**22**	Number of flowers/plant	9.5±1.58	11±2.16	12.2±1.0
**Flower**				
**23**	Petal size (mm)	4.77±0.59	4.06±0.8	4.15±0.91
**24**	Sepal size (mm)	3.01±0.39	2.99±0.40	3.38±0.5
**Pollen size**				
**25**	Pollen size (µm)	37.24±1.15×39.06±0.79	41.05 ± 0.52×44.55 ± 0.59	45.24±0.57×46.19±0.38

### B) Cytological observations

**Figure 2.**
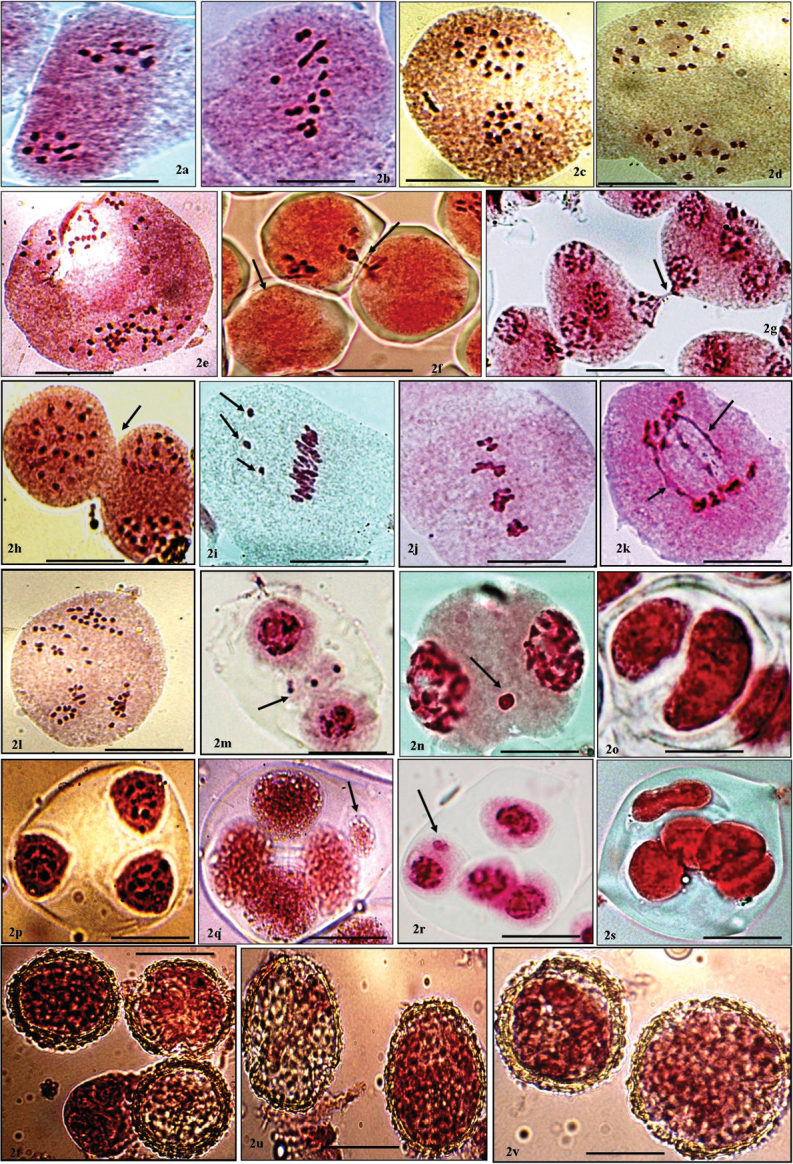
**a** (Diploid cytotype) PMC at Anaphase-I showing 8:8 distribution of chromosomes **b** (Diploid cytotype) PMC at Metaphase-I showing 8II bivalents **c** (Tetraploid cytotype) PMC at Anaphase-I showing 16:16 distribution of chromosomes **d** PMC at Metaphase-II showing 16:16 distribution of chromosome **e** (Octaploid cytotype) PMC at Anaphase-I showing 32:32 distribution of chromosomes **f** (Diploid cytotype) Three PMCs showing transfer of chromatin material (arrowed) **g** A group of PMCs (arrowed) involved in cytomixis at telophase-I **h** (Tetraploid cytotype) Size difference and direct connection between two PMCs during chromatin material (arrowed) **i** PMC at metaphase-I showing unoriented bivalent (arrowed) **j** PMC at metaphase-I showing chromatin stickiness **k** PMC at anaphase-I showing chromatin bridges (arrowed) **l** PMC at anaphase-II showing unequal distribution of chromosomes (arrowed) **m–n** PMC at telophase-I showing chromatin chromosomal laggards (arrowed) **o** Diad **p** Triad **q–r** Tetrad with micronuclei (arrowed) **s** Polyad **t–v** Fertile, sterile and Heterogeneous sized fertile pollen grains. Bar = 10 µm.

Based on x=8 (Darlington and Wyile 1955), the 22 different populations of *Valeriana jatamansi* examined for cytological variations revealed the existence of diploid (2n=16), tetraploid (2n=32) and octaploid (2n=64) cytotypes. Out of 22 populations, two diploid (2n=16) populations showing 8:8 distribution of chromosomes at anaphase-I (Fig. 2a, b) are chromosomally reported for the first time from India. However, diploid (2n=16) cytotypes have been previously reported from Germany and Pakistan by Engel (1974), Khatoon and Ali (1993), respectively. The occurrence of eighteen tetraploid cytotypes with 16:16 distribution of chromosomes at anaphase-I (Fig. 2c, d) and is in conformity with the earlier reports from Kashmir Himalayas (Jee et al. 1983). Two other populations with 2n=64 (32: 32 distribution of chromosomes at anaphase-I) (Fig. 2e) have been cytologically worked out for the first time on worldwide basis.

A perusal of cytological literature reveals that majority of the species in the genus have been worked out showing 2n=14, 16, 28, 32, 42, 48, 56, 60, 64, 72, 80, 90 and 96. The genus is dibasic with x=7, 8 as has been suggested by Darlington and Wylie (1955). The highest level of ploidy of the genus has been reported to be 12x (Engel 1976).

### C) Geographical distribution

**Figure 3.**
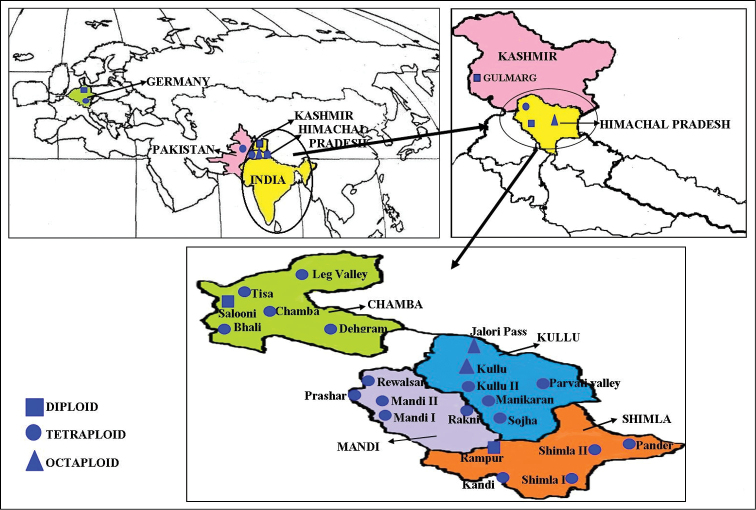
Geographical distribution of 2x, 4x and 8x cytotypes of *Valeriana jatamansi*.

In the North-Western Himalayas, the distribution pattern of euploid cytotypes shows definite relation to altitudinal variations (Table 1). The distribution of diploids in Himachal Pradesh is uncommon but some accessions are available from lower altitudes of 1350–1900 m. Tetraploids are the most common and are widely distributed within the altitude of 764–2236 m in the state. The octaploid cytotypes are restricted only to higher altitude localities (3,164–3,647 m) in Kullu district of Himachal Pradesh. Thus it is clear that north-western Himalayan region harbours maximum genetic diversity for the species (Fig. 3).

### Meiotic abnormalities

The meiotic course was found to be abnormal in eight populations (Table 3). In such populations, abnormalities in the form of cytomixis, unoriented bivalents, chromatin stickiness, chromatin bridges and laggards, or multipolarity have been observed at different stages of meiosis (Fig. 2f–s; Table 3). Thus in the presently studied populations indicate the existence of intraspecific genetic diversities in the species. Such genetic differences have earlier been reported in different plant species (Sheidai et al. 2003, Rani et al. 2013, Jeelani et al. 2013). The phenomenon of chromatin transfer from early prophase to the pollen formation stage (Fig. 2f–h) has been observed in most of these populations with the highest percentage recorded in populations P-20 from Kullu II (Table 3).

**Table 3.** Data on cytomixis, abnormal meiotic course and pollen fertility in *Valeriana jatamansi*.

	Cytomixis at Meiosis-I/ Meiosis II		Meiotic course showing PMCs with				Pollen grains
Population number	% of PMCs involved	Number of PMCs Involved	Chromosomal stickiness at M-I (%)	Bridges at Meiosis-I/ Meiosis-II (%)	Laggards at Meiosis-I/ Meiosis-II (%)	Micronuclei at T-II (%)	Fertility (%)
PP-1	8.33 (10/120)/	2-3	4.00 (4/100)	5.88 (6/102)/	2.86 (2/70)/	17.47 (18/103)	66.23
	7.07 (7/99)			-	2.63 (2/76)		
PP-3	5.78 (7/121)/	2-3	-	3.80 (4/105)/	2.70 (2/74)/	18.18 (16/88)	65.07
				-	2.66 (2/75)		
PP-6	4.34 (5/115)/	2-3	-	2.40 (3/125)/	3.30 (4/121)/	5.78 (7/121)	75.78
	4.59 (4/87)			1.73 (2/115)	-		
PP-10	-	-	4.87 (6/123)	2.27 (3/132)/	-	7.57 (10/132)	70.32
				2.60 (3/115)			
PP-14	5.88 (6/102)/	2-4	9.75 (12/123)	5.35 (6/112)/	5.69 (7/123)/	4.46 (5/112)	71.65
	6.25 (7/112)			3.92 (4/102)	4.83 (6/124)		
PP-16	-	-	7.33 (8/109)	-	3.53 (4/113)/	-	77.87
					-		
PP-17	-	-	8.33 (11/132)	2.52 (3/119)/	-	5.60(7/125)	79.67
				-			
PP-20	9.00 (9/100)/	2-4	-	-	5.73 (7/122)/	4.50(5/111)	79.65
	8.00 (10/125)				-		

Cytomixis in these populations resulted in the production of hyper- and hypo-ploid PMCs. According to Sarvella (1958), cytomixis results in some genetic consequences and it is a mechanism to explain the origin of aneuploid gametes. Some others considered it to be of great importance as the most probable consequence of cytomixis is the formation of hypo-, hyperploid and enucleated PMCs, aberrant microspore tetrads and pollen sterility (Sheidai and Fadaei 2005, Jeelani et al. 2011). Cytomixis results in the production of unreduced gametes in several angiosperms and leads to the production of aneuploid or polyploids plants (Fadaei et al. 2010, Mandal et al. 2013). The formation of unreduced gametes is of evolutionary significance in a way that it can lead to the production of plants with higher ploidy level through polyploidization (Villeux 1985). Chromatin stickiness involving few bivalents or whole complement was seen from prophase-I to metaphase-I (Fig. 2i–j). Cytomixis and chromatin stickiness are considered to be the result of genetic factors (Bellucci et al. 2003, Ghaffari 2006, Fadaei et al. 2010) and environmental factors (Nirmala and Rao 1996) as well as genomic–environmental interaction (Baptista-Giacomelli et al. 2000) and seems to be equally applicable to the presently investigated populations. Chromatin stickiness also results in the formation of fragmented chromatin (Kumar and Singhal 2011) and at present very low percentage of such fragments was observed.

During present investigations, single or multiple bridges (Fig. 2k) have been recorded in six populations with highest percentage in Population -14 from Prashar. According to Rothfels and Mason (1975) bridges may originate from chiasma formation in heterozygous inversions. Bridges and fragments are the results of spontaneous breakage and fusion of the chromosomes. Early disjunction of bivalents normally does not affect the normal distribution of chromosomes at anaphase-I, but late separation of bivalents which normally exists in hybrids and cytologically abnormal diploids causes some meiotic disturbances (chromatin bridges and laggards) and consequently pollen malformation (Wang et al. 2004). Bivalents and chromosomes that lag behind and are unable to reach at poles during anaphase-I, telophase-I, anaphase- II and telophase-II stages of meiosis form laggards. In our study, chromosomal laggards were noticed in six populations (Fig. 2l–n). There are different explanations for the formation of chromosomal laggards such as interlocking of bivalents and paracentric inversions (Sinha and Godward 1972, Tarar and Dyansagar 1980). One of the most acceptable reasons for the formation of chromosomal laggards is lack of synapsis at early prophase stages or precocious separation and delayed terminalization of chiasmata (Pagliarini 1990, Kumar and Tripathi 2007). All these meiotic abnormalities result in abnormal microsporogenesis, leading to the formation of monads, dyads, triads, or polyads (Fig. 2o–s; Table 4). Furthermore, micronuclei have also been observed in most of these species (Fig. 2q–r; Table 4). These meiotic abnormalities along with abnormal microsporogenesis lead to the formation of heterogeneous sized (large and small) fertile pollen grains and reduced pollen fertility (Fig. 2t–v). The frequency of large-sized pollen grains ranges from 3% to 4% in different populations. The occurrences of large pollen grains conforms to previous information about possibility of such pollen grains to be resulting from unreduced 2n gametes as has been seen in several angiosperms (Bertagnolle and Thomson 1995, Sheidai et al. 2008, Fadaei et al. 2010, Jeelani et al. 2011). Presence of genetic diversity within the populations of *Valeriana jatamansi* stresses the need for further cytological analysis from different geographical areas. Intraspecific variability at population level has been brought to the fore, to be conserved and/or utilized for further plant improvement programme. The genetic diversity in *Valeriana jatamansi* points towards the need of studies for chemical and molecular characterization and biological activity of these variants to identify superiour chemotypes for further conservation and exploitation.

**Table 4.** Data on abnormal microsporogenesis in different cytotypes of Valeriana jatamansi marked with abnormal meiosis.

Microsporogenesis Accessions								
	Dyads		Triads		Tetrads		Polyads	
	WMN	WN	WMN	WN	WMN	WN	WMN	WN
PP-1	0.99 (1/101)		0.99 (1/101)	1.98 (2/101)	71.28 (72/101)	21.78 (22/101)	1.98 (2/101)	-
PP-3	-	-	2.04 (2/98)	-	76.53 (75/98)	21.42 (21/98)	-	-
PP-6	0.96 (1/104)	-	0.96 (1/104)	-	70.19 (73/104)	26.92 (28/104)	0.96 (1/104)	-
PP-10	2.04 (2/98)	1.02 (1/98)	2.04 (2/98)	-	88.77 (87/98)	6.12 (6/98)	-	-
PP-14	2.97 (3/101)	0.99 (1/101)	3.96 (4/101)	0.99 (1/101)	79.20 (80/101)	9.90 (10/101)	0.99 (1/101)	0.99 (1/101)
PP-16	-	0.94 (1/106)	2.83 (3/106)	0.94 (1/106)	84.90 (90/106)	10.37 (11/106)	-	-
PP-17	-	-	0.98 (1/102)	0.98 (1/102)	89.21 (91/102)	8.82 (9/102)	-	-
PP-20	1.86 (2/107)	0.93 (1/107)	3.73 (4/107)	0.93 (1/107)	76.63 (82/107)	14.01 (15/107)	0.93 (1/107)	0.93 (1/107)

WMN = without micronuclei; WM = with micronuclei.
